# Sodium Channel–Mediated Ventricular Arrhythmia After *Delphinium denudatum* Ingestion Managed Conservatively in a Resource-Limited Setting

**DOI:** 10.1016/j.jaccas.2026.107164

**Published:** 2026-02-26

**Authors:** Nirdosh Rassani, Ken Grauer

**Affiliations:** a Aria Institute of Medical Sciences, Quetta, Pakistan; b College of Medicine, University of Florida, Gainesville, Florida, USA

**Keywords:** electrophysiology, tachycardia, ventricular tachycardia

## Abstract

**Background:**

*Delphinium denudatum* (jadwār) is a traditional South Asian herbal root containing aconitine-like alkaloids that cause sodium channel–mediated cardiotoxicity. Human toxicity data remain limited, particularly from resource-limited settings.

**Case Summary:**

A man in his late 20s presented 6 hours after accidental ingestion of *Ddenudatum* root with palpitations and autonomic symptoms. Continuous electrocardiographic monitoring revealed frequent ventricular ectopy with nonsustained polymorphic ventricular tachycardia. He was treated with intravenous lidocaine and magnesium sulfate, resulting in rapid suppression of ventricular arrhythmias. Lidocaine infusion was continued for 24 hours given uncertain toxin pharmacokinetics, followed by extended telemetry observation.

**Discussion:**

This case demonstrates mechanism-based antiarrhythmic therapy for aconitine-like poisoning and highlights the importance of prolonged monitoring given the risk of delayed malignant arrhythmias.

**Take-Home Message:**

Early sodium-channel blockade with lidocaine and structured cardiac monitoring can be lifesaving in plant-based cardiotoxicity.

## Graphical Abstract

**Figure:**
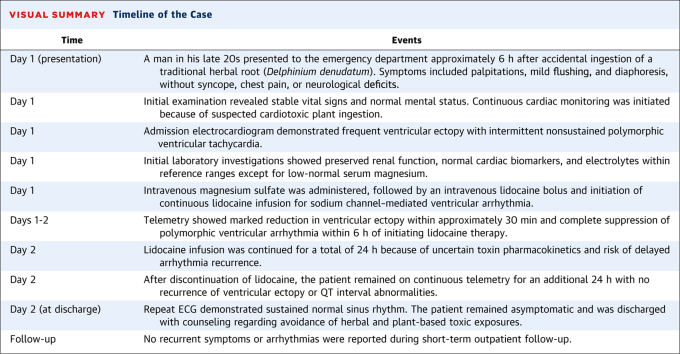

## Background

Intoxication with diterpenoid alkaloids derived from *Delphinium* and *Aconitum* species is rare in Western countries but remains a clinically relevant problem in many parts of Asia, where plant-based toxins are frequently implicated in both accidental and intentional poisonings.^1^ Veratridine- and aconitine-containing plants are well-recognized causes of life-threatening ventricular arrhythmias and are among the commonly reported plant poisons in South and East Asia, particularly in rural settings.^2^ Although population-level incidence data are limited, regional toxicology series consistently report sodium channel–activating plant alkaloids as an important cause of malignant arrhythmias and sudden death.^3^ In Pakistan and neighboring countries, *Delphinium denudatum* (jadwār) continues to be used as a traditional remedy, underscoring the ongoing clinical relevance of such intoxications in resource-limited environments.

**Take-Home Message**

• Early sodium-channel blockade with lidocaine and structured cardiac monitoring can be lifesaving in plant-based cardiotoxicity.

**Figure 1:**
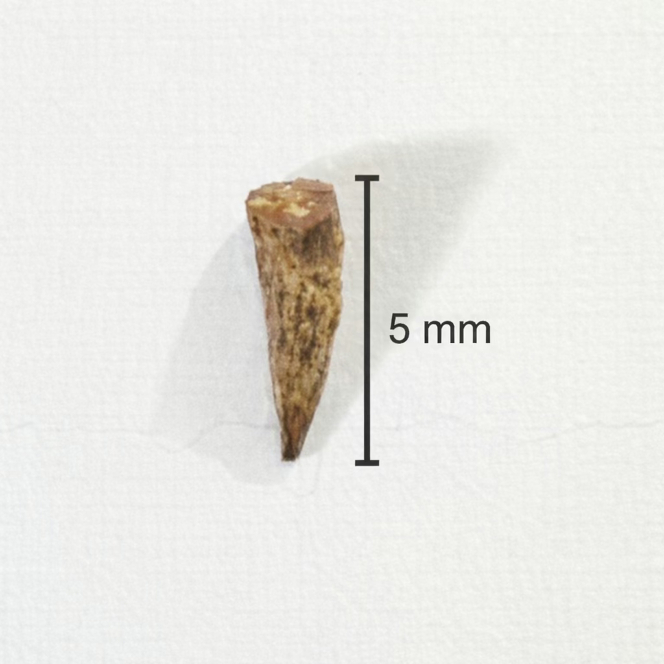
*Delphinium denudatum* (Jadwar) Photograph of *Delphinium denudatum*, commonly known as jadwār, a traditional herbal root widely used in South Asia for analgesic, anxiolytic, and cultural or “spiritual” indications. The plant root was implicated in the present case.

We report a case of accidental ingestion of *D* *denudatum* root (Figure 1) resulting in early autonomic manifestations and monitored management with favorable outcome.

## History of Presentation

A man in his late 20s with a history of diabetes mellitus accidentally ingested approximately a finger-breadth piece of *D* *denudatum* root. He presented to the emergency department approximately 6 hours postingestion with reports of mild flushing, increased sweating, and palpitations. There was no vomiting, syncope, chest pain, or neurological deficits.

### Initial Examination

Examination findings were as follows:

• Glasgow coma scale: 15/15
• Vital signs: blood pressure 131/67 mm Hg, heart rate 78 beats/min, respiratory rate 16 breaths/min, peripheral oxygen saturation 98% on room air, temperature 36.7 °C
• Pupils: 2 mm, bilaterally equal and reactive
• Chest/Abdomen: clear; soft, nontender
• Neurological examination: normal
• No paresthesias, convulsions, or respiratory distress

## Past Medical History

The patient had a history of type 2 diabetes mellitus, with no known cardiac disease or prior arrhythmias. He was not taking any regular medications related to QT prolongation.

## Differential Diagnosis

Considerations included aconitine-like alkaloid poisoning (*Delphinium* species), acute coronary syndrome, primary electrical disease (eg, channelopathy), electrolyte-mediated ventricular arrhythmia, and myocarditis.

## Investigations

Initial laboratory investigations demonstrated a hemoglobin level of 14.5 g/dL, white blood cell count of 9.8 × 10^9^/L, and platelet count of 187 × 10^9^/L, all within normal reference ranges. Inflammatory markers showed a mildly elevated C-reactive protein of 10 mg/L. Cardiac biomarkers were negative, with a high-sensitivity troponin T level of 7 ng/L (reference: <14 ng/L). Renal function was preserved, with a serum creatinine of 0.6 mg/dL and urea of 45 mg/dL. Electrolyte evaluation revealed normal serum calcium (9.2 mg/dL) and potassium (4.3 mmol/L). Serum bicarbonate was at the lower limit of normal at 21.2 mmol/L. Serum magnesium was initially at the lower limit of normal (1.6 mg/dL) and increased to 1.8 mg/dL after administration of a 2-g intravenous magnesium sulfate bolus.

Echocardiography showed normal-sized cardiac chambers and a normal-sized left ventricle with normal systolic function and an ejection fraction of 60%. E/A fusion due to tachycardia was indicated. The right ventricle was normal sized with normal function. Mild tricuspid regurgitation and mild pulmonary hypertension were seen, with no clots or vegetations.

## Management

Recognizing the risk of aconitine-like sodium-channel activation and malignant ventricular arrhythmias, the patient was immediately placed on continuous cardiac monitoring. Because the ingestion timing and toxin profile suggested significant arrhythmic risk, he was administered intravenous lidocaine, which competitively blocks open sodium channels and counteracts aconitine toxicity.

All interventions and therapies administered are listed in Table 1. Other supportive medications (atropine, lipid emulsion) were kept readily available at the patient's bedside but were not given.

**Table 1:** Therapies Administered

Intervention	Dosage and Duration
Lidocaine bolus	100 mg intravenous emergently
Lidocaine infusion	4 mg/min for 24 h
Magnesium sulfate	2 g
Monitoring	Continuous ECG and vitals
Supportive care	Intravenous fluids, close neurocardiac observation

ECG = electrocardiogram.

## Outcome and Follow-Up

**Figure 2:**
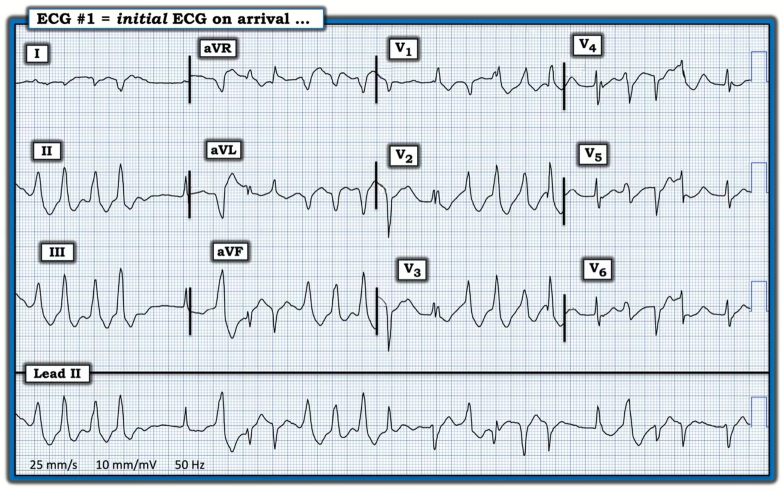
Admission Electrocardiogram 12-lead electrocardiogram obtained on arrival demonstrating an irregularly irregular rhythm without discernible P waves, consistent with atrial fibrillation. The rhythm strip (lead II) shows intermittent wide-complex beats representing nonsustained runs of polymorphic ventricular tachycardia. Narrow-complex beats exhibit beat-to-beat variation in QRS morphology, suggestive of aberrant conduction and/or fusion with ventricular ectopy. Simultaneously recorded lateral leads (V5 and V6) demonstrate ST-segment depression with associated QT prolongation. ECG = electrocardiogram. Image digitized using PMCardio.

The patient remained hemodynamically stable throughout the hospitalization. During the initial period of continuous telemetry, frequent ventricular ectopy with short runs of nonsustained polymorphic ventricular tachycardia (VT) were observed, consistent with aconitine-type sodium channel toxicity (Figure 2).

After administration of an intravenous lidocaine bolus, a clear therapeutic effect was noted: Within approximately 30 minutes, the frequency and duration of nonsustained polymorphic VT and ventricular ectopy decreased, supporting a drug-related antiarrhythmic response rather than spontaneous resolution. Lidocaine was subsequently continued as a continuous infusion. Complete suppression of ventricular ectopy was achieved by approximately 6 hours after the initiation of lidocaine therapy.

Despite early electrical quiescence, the lidocaine infusion was intentionally maintained for a total of 24 hours, given the absence of established pharmacokinetic data and undefined elimination half-life of *D* *denudatum* alkaloids, as well as the recognized risk of delayed malignant ventricular arrhythmias in aconitine-like poisonings.

**Figure 3:**
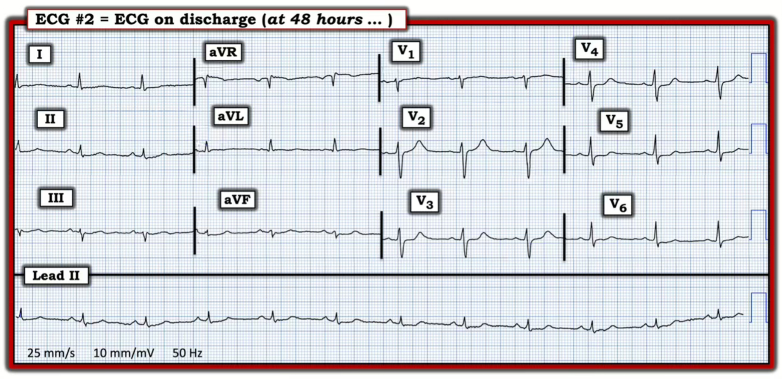
Discharge Electrocardiogram at 48 Hours After Admission Follow-up electrocardiogram showing restoration of normal sinus rhythm at 65 beats/min with normal PR, QRS, and corrected QT intervals and a mildly leftward frontal plane axis (–10°). Diffuse low voltage is present with nonspecific ST-segment flattening and mild ST-segment depression in multiple leads, along with subtle ST-segment elevation in lead aVR, consistent with residual subendocardial ischemia. Overall, there is marked improvement compared with the admission tracing. ECG = electrocardiogram. Image digitized using PMCardio.

After completion of the 24-hour lidocaine infusion, the patient was observed on continuous telemetry for an additional 24 hours. No recurrence of ventricular ectopy, QT interval abnormalities, or neurological symptoms was noted during this postinfusion observation period. A repeat ECG in the ED after 48 hours confirmed sustained normal sinus rhythm without residual ectopy (Figure 3). The patient remained asymptomatic and was discharged safely with counseling regarding the risks of herbal and plant-based toxic exposure.

This time-linked clinical response supports a management strategy of early sodium-channel blockade with lidocaine, continuation through confirmed arrhythmic quiescence, and extended monitoring for relapse in poisonings with poorly defined toxicokinetics.

## Discussion

### Pathophysiology

*D* *denudatum* (jadwār) contains diterpenoid aconitine-type alkaloids that bind the open state of cardiac voltage-gated Na^+^ channels (Na_V_1.5), markedly slowing inactivation and causing persistent inward Na^+^ current.^4^, ^5^, ^6^ This produces prolonged phase 0/plateau depolarization, intracellular Na^+^/Ca^2+^ overload, early and delayed afterdepolarizations, and triggered activity—a substrate for ventricular ectopy, polymorphic VT, and ventricular fibrillation (VF).^7^ Secondary effects on K^+^ and Ca^2+^ currents may further destabilize repolarization. Autonomic swings (initial vagotonia followed by sympathetic surge) can amplify arrhythmogenicity.

Aconitine has historically served as one of the earliest experimental models for studying cardiac arrhythmogenesis. As early as the mid-20th century, aconitine was used to reproducibly induce ventricular arrhythmias through impairment of sodium-channel inactivation, providing foundational insights into triggered activity and re-entrant mechanisms.^3^^,^^8^ This experimental approach remains relevant today, with aconitine still employed in contemporary electrophysiology research to model sodium-channel–mediated arrhythmias and test antiarrhythmic strategies.

### Clinical phenotype

Cardiac toxicity is the predominant clinical feature, characterized by ventricular ectopy progressing to VT/VF, hypotension due to reduced myocardial contractility and vasodilation, and occasionally bradyarrhythmias or atrioventricular block. Neurologic symptoms (paresthesias, perioral tingling, dizziness) and gastrointestinal upset may occur. ECG findings are variable, ranging from frequent premature ventricular complexes to malignant polymorphic VT, with QTc often normal or modestly prolonged (true torsadogenic physiology is less typical than triggered activity from Na^+^ channel dysfunction).

### Rationale for antiarrhythmic choice

Lidocaine (class Ib) exhibits use-dependent block of open/inactivated Na^+^ channels, shortening action potential in depolarized/ischemic tissue and suppressing triggered activity caused by aconitine-type toxins.^9^ These properties make it the first-line agent in many reports of aconite-like poisonings. Procainamide (class Ia) is a reasonable alternative/adjunct in refractory cases.

Amiodarone, although widely trusted in clinical practice, may be suboptimal in aconitine-like poisonings. Its class I (sodium-channel blocking) effects are known to develop slowly and are markedly delayed compared with pure class Ib agents. This delayed onset may limit its utility in rapidly evolving toxin-induced ventricular arrhythmias, where early suppression of persistent sodium current is critical.^10^ Furthermore, its additional beta-blocking and calcium channel–blocking properties may exacerbate hypotension or bradyarrhythmias in poisoned patients. These pharmacodynamic considerations further support lidocaine or procainamide as preferred first-line agents in this context.

Magnesium sulfate is appropriate for documented torsades de pointes (rare in this toxidrome) but is not routinely indicated. Electrical cardioversion-defibrillation remains indicated for unstable VT/VF. Overdrive pacing or extracorporeal membrane oxygenation are salvage options in refractory malignant arrhythmias.^11^

### Decontamination and supportive care

If ingestion is early and secure airway is ensured, single-dose activated charcoal can be considered (plant alkaloids may undergo enterohepatic recirculation). Gastric lavage is rarely indicated and only with expert input. Optimize K^+^ and Mg^2+^ (high-normal targets), correct acidosis, and treat hypotension with balanced fluids and vasopressors as needed.

Regarding sodium bicarbonate, routine alkalinization is not recommended; consider only if there is QRS widening or clinically significant metabolic acidosis, otherwise it does not counter the primary toxin mechanism.

### Monitoring strategy and duration

Given the uncertain pharmacokinetics of *D* *denudatum* alkaloids owing to variability in species, plant part ingested, preparation method, and the presence of multiple coalkaloids, continuous cardiac monitoring for 24 to 48 hours is prudent. In the present case, ventricular ectopy began to attenuate within 30 to 40 minutes after an intravenous lidocaine bolus, supplemented with 2 g of magnesium sulfate. Complete suppression of ventricular arrhythmia was achieved approximately 6 hours after initiation of lidocaine therapy. Despite early arrhythmic quiescence, the lidocaine infusion was deliberately continued for a total of 24 hours in view of the risk of delayed or recurrent dysrhythmias associated with alkaloids of undefined elimination half-life. Prolonged observation thereafter revealed no recurrence of ectopy, and ECG after 48 hours confirmed sustained normal sinus rhythm.

Overall, this clinical course supports a pragmatic strategy of “treat until electrical quiescence, then observe for relapse” in toxin-induced arrhythmias where pharmacokinetic parameters are poorly characterized.

### Implications for resource-limited settings

Where herbal remedy use is common and advanced cardiotoxicant care (temporary pacing/extracorporeal membrane oxygenation) is scarce, a protocolized pathway—early recognition, charcoal when safe, first-line lidocaine, electrolyte optimization, cautious avoidance of amiodarone, and telemetry for 24 to 48 hours—is pragmatic and reproducible. This case also underscores the value of history-taking with plant identification (photo/physical specimen) to direct antidysrhythmic selection.

### Study limitations

A key limitation of this report is the absence of pharmacokinetic characterization of *D* *denudatum* alkaloids in humans. As a result, conclusions regarding the efficacy and optimal duration of lidocaine therapy remain inferential and based on mechanistic rationale rather than direct toxin-drug interaction data.

## Conclusions

This case of *D* *denudatum* poisoning manifested with ventricular ectopy consistent with aconitine-like Na^+^ channel toxicity, and it was successfully managed with intravenous lidocaine (100 mg bolus→4 mg/min infusion for 24 hours), meticulous electrolyte control, and continuous monitoring. Ectopy resolved within 6 hours, no malignant arrhythmias emerged, and ECG after 48 hours confirmed durable sinus rhythm. In regions with prevalent herbal exposures and limited rescue capacity, early toxin mechanism–matched therapy (class Ib Na^+^ block) plus structured observation can prevent catastrophic ventricular arrhythmias and enable safe discharge.

### Ethics Statement

This case report was conducted in accordance with institutional ethical standards and the principles outlined in the Declaration of Helsinki. Formal institutional review board approval was not required for publication of a single anonymized case report.

## Funding Support and Author Disclosures

The authors have reported that they have no relationships relevant to the contents of this paper to disclose.

**Visual Summary:** Timeline of the Case

Time	Events
Day 1 (presentation)	A man in his late 20s presented to the emergency department approximately 6 h after accidental ingestion of a traditional herbal root (*Delphinium denudatum*). Symptoms included palpitations, mild flushing, and diaphoresis, without syncope, chest pain, or neurological deficits.
Day 1	Initial examination revealed stable vital signs and normal mental status. Continuous cardiac monitoring was initiated because of suspected cardiotoxic plant ingestion.
Day 1	Admission electrocardiogram demonstrated frequent ventricular ectopy with intermittent nonsustained polymorphic ventricular tachycardia.
Day 1	Initial laboratory investigations showed preserved renal function, normal cardiac biomarkers, and electrolytes within reference ranges except for low-normal serum magnesium.
Day 1	Intravenous magnesium sulfate was administered, followed by an intravenous lidocaine bolus and initiation of continuous lidocaine infusion for sodium channel–mediated ventricular arrhythmia.
Days 1-2	Telemetry showed marked reduction in ventricular ectopy within approximately 30 min and complete suppression of polymorphic ventricular arrhythmia within 6 h of initiating lidocaine therapy.
Day 2	Lidocaine infusion was continued for a total of 24 h because of uncertain toxin pharmacokinetics and risk of delayed arrhythmia recurrence.
Day 2	After discontinuation of lidocaine, the patient remained on continuous telemetry for an additional 24 h with no recurrence of ventricular ectopy or QT interval abnormalities.
Day 2 (at discharge)	Repeat ECG demonstrated sustained normal sinus rhythm. The patient remained asymptomatic and was discharged with counseling regarding avoidance of herbal and plant-based toxic exposures.
Follow-up	No recurrent symptoms or arrhythmias were reported during short-term outpatient follow-up.
